# Editorial: Cellular and molecular mechanisms that govern assembly, plasticity, and function of GABAergic inhibitory circuits in the mammalian brain

**DOI:** 10.3389/fncel.2025.1568845

**Published:** 2025-02-11

**Authors:** Yasufumi Hayano, Goichi Miyoshi, Anirban Paul, Hiroki Taniguchi

**Affiliations:** ^1^Department of Pathology, The Ohio State University Wexner Medical Center, Columbus, OH, United States; ^2^Chronic Brain Injury Program, The Ohio State University Wexner Medical Center, Columbus, OH, United States; ^3^Department of Developmental Genetics and Behavioral Neuroscience, Gunma University Graduate School of Medicine, Maebashi, Japan; ^4^Department of Neural and Behavioral Sciences, College of Medicine, Pennsylvania State University, Hershey, PA, United States

**Keywords:** inhibitory interneuron, GABA, neurodevelopmental disorder, cell type, circuit assembly, neuronal circuit

Inhibitory regulations by GABAergic interneurons (INs) play an essential role in intricate neural computations in normal brains, and their malformation and malfunction lead to a variety of brain disorders (Del Pino et al., 2018; Frye et al., 2016; Kepecs and Fishell, 2014; Takano, 2015; Taniguchi, 2014; Tremblay et al., 2016; Wang et al., 2016). Over the past two decades, there has been a remarkable progress in understanding development, plasticity, function, and pathological relevance of GABAergic inhibitory circuits. In particular, along with recent rapid technological advances in single-cell omics, genetic targeting, *in vivo* imaging, functional manipulations, and behavioral assays, our knowledge on IN subtypes has been explosively expanded. A Research Topic of articles including seven original research papers and two reviews, organized under the theme “Cellular and Molecular Mechanisms that Govern Assembly, Plasticity, and Function of GABAergic Inhibitory Circuits in the Mammalian Brain,” highlights just how far we've come—and where we need to go next. These reports comprehensively discuss topics on the GABAergic inhibitory system ranging from cell type specification, synaptic assembly, and functional diversity to its role in health and disease. The overarching goal is to untangle how myriad of INs weave themselves into functional circuits—a puzzle central to understanding the power and vulnerability of cortical inhibition.

The challenging but essential tasks for dissecting the inhibitory system is to disentangle intricate inhibitory circuits consisting of diverse GABAergic IN subtypes (Bandler et al., 2017; Hu et al., 2017; Lodato and Arlotta, 2015; Miyoshi, 2019; Pelkey et al., 2017). Machold and Rudy review the emerging view on cortical and hippocampal IN subtypes defined by transcriptomics and developmental origin and highlight a genetic toolkit for targeting specific IN subtypes, along with the technical considerations inherent to each approach. The authors provide gene expression heatmaps illustrating transcriptomic identity of cortical and hippocampal IN subtypes as well as a table summarizing Cre/Flp driver lines and Cre/Flp-dependent reporter lines for investigating IN subtypes that are currently available. Expanding genetic toolkit allowing for targeting more specific IN subtypes will further deepen our understanding of IN specification, assembly, and function and facilitate gaining novel insight into brain diseases. Yet, pinpointing these subtypes is only half the story—each interneuron lineage arises from a dynamic mix of genetic and epigenetic codes where the interplay of chromatin modifiers can direct interneuron destinies, reshaping the fundamental map of inhibitory identity.

It is fundamental to ask how genetic and epigenetic regulators orchestrate generation and specification of inhibitory cell types, and Rhodes et al. demonstrate that loss of histone methyltransferase Ezh2 in the medial ganglionic eminence (MGE) disrupts H3K27me3 levels, leading to significant changes in interneuron fate, with increased somatostatin-expressing (SST+) INs and decreased parvalbumin-expressing (PV+) INs, indicating that the MGE is not a uniform source of PV+ INs and SST+ INs simply marching along a preordained path. Instead, these progenitors rely on nuanced chromatin remodeling to steer them toward particular fates. By perturbing Ezh2, the authors reveal how epigenetic mechanisms bias lineage outcomes, ultimately capable of altering interneuron composition and circuit-level consequences. This aligns with a growing literature showing that interneuron progenitor domains—from MGE to CGE—are not static templates, but dynamic entities whose transcriptional and epigenetic landscapes are continuously shaped by internal gene regulatory networks and external signaling cues.

While epigenetic codes inscribe interneuron destinies, GABA itself can orchestrate a variety of developmental maturational processes in the brain such as neuronal migration, synapse formation, neurite elongation, and circuit integration (Bortone and Polleux, 2009; Kilb, 2021; Peerboom and Wierenga, 2021). GABA_A_ receptors (GABA_A_Rs) and K-Cl transporter 2 (KCC2) play a major role in this regulation. Zavalin et al. tracks the developmental expression patterns of GABA_A_R subunits and KCC2. The discovery of region- and layer-specific changes in receptor composition during early postnatal maturation exemplifies how the inhibitory system is constructed in phases—akin to a building whose scaffolding and wiring are put in place step-by-step rather than all at once. This temporal and spatial refinement is pivotal for stabilizing nascent circuits, ensuring that as excitatory inputs proliferate and refine, the inhibitory networks are calibrated in lockstep, achieving a balanced interplay that underlies the cortex's computational prowess.

Development and maturation of inhibitory synapses are key cellular processes to establish functional GABAergic synaptic transmission in neuronal networks. Establishing robust inhibitory synapses requires an additional layer of coordination—a molecular dialogue at the synapse itself. Here Sui et al. reveals the synergy between Neuroligin-2 (NL2) and GABA_A_Rs and how their conversation dictates inhibitory circuit assembly. NL2 and GABA_A_Rs, inhibitory postsynaptic cell surface proteins, have been demonstrated to synergistically recruit inhibitory synapses in a heterologous co-culture system containing HEK cells and striatal GABAergic medium spiny neurons (Fuchs et al., 2013). Sui et al. extend this finding by investigating the effect of different types of GABA_A_Rs on NL2 synaptogenic activity using the same assay system as well as conducting structure/function analysis of GABA_A_Rs. The authors find that the synaptic type GABA_A_Rs (α2β2γ2-GABA_A_Rs) have a significantly greater effect in facilitating the NL2-dependent induction of synapses than the prototypical extrasynaptic type GABA_A_Rs (α4β3δ-GABA_A_Rs). They show that this synergistic effect of GABA_A_Rs on NL2-dependent inhibitory synapse recruitment is independent of GABA_A_R channel activity. Furthermore, they demonstrate that the synergism between GABA_A_Rs and NL2 is dependent on the γ2 subunit interaction with NL2, and the intracellular domain of this subunit is necessary for this interaction. These findings reveal the molecular logic underlying the GABA_A_Rs and NL2 interaction that mediates morphological and functional coordination in inhibitory synapse development.

Finely-tuned synaptic elements modulate circuit activity across behavioral contexts, reflecting a dynamic dialogue between interneurons and the overall brain state—a hallmark of GABAergic control. However, much is not known about the cortical mechanisms behind the modulation of neuronal activity across behavioral states. Sabri and Batista-Brito, utilizing chemogenetics and multi-cell spike recording, demonstrate that inhibiting vasoactive intestinal peptide (VIP)-expressing (VIP+) INs throughout the brain reduces the correlation between the mouse facial motion and the spiking activity of individual neurons in the primary visual cortex. The authors also find that inhibiting VIP+ INs during the quiet state results in enhanced slow rhythms while reducing fast spike synchrony. Their findings suggest that VIP interneurons modulate cortical activity in a behavior-dependent manner across different behavioral states.

Even beyond moment-to-moment states, key transcription factors guide the balance between excitation and inhibition over the course of development, and small disruptions in this orchestration can reverberate into neurodevelopmental disorders, as we now observe. Myocyte enhancement factor 2c (MEF2C), a transcription factor expressed in both excitatory PNs and inhibitory INs throughout the life in mice, have been implicated in various neurodevelopmental disorders (NDDs) such as autism spectrum disorder (ASD), schizophrenia, and bipolar disorder (Assali et al., 2019; Harrington et al., 2016; Rajkovich et al., 2017; Tu et al., 2017). In a minireview, Ward et al. review how MEF2C loss-of-function (LOF) impacts excitatory and inhibitory cortical circuit development and highlight how brain dysfunction and altered behavior may derive from the dysfunction of specific cortical circuits at specific developmental times along with a table showing cellular and behavioral phenotypes in MEF2C LOF mouse models. These more nuanced studies in MEF2C LOF mice could provide a suggestive hint for identifying prognostic biomarkers and developing early intervention in NDDs.

As outlined by the above review, a mutation in one risk factor gene for NDDs could differentially impact development and functional maturation of distinct cell types and neuronal circuits, and thus distinct behavioral deficits in NDDs that involve dysfunction in different sets of circuit modules may be attributable to separate pathological landscapes. Asano et al. address this line of question using the ASD mouse model with FOXG1 haploinsufficiency. The authors find that in the ASD model, while social behavior deficits are evident from the early juvenile stages, novel space preference is initially established during early juvenile stages but regresses by postnatal week 6. Furthermore, they demonstrate that in contrast to their previous finding that reducing GABAergic tone decreased social scores in wildtype mice and exacerbated social deficits in the ASD model (Miyoshi et al., 2021), this reduction has no impact on novel space preference in either wildtype or ASD model mice. This dissociation underscores that the relationship between developmental inhibitory dysfunction and resulting behavioral phenotypes is not one-size-fits-all. Different aspects of behavior and cognition may rely on distinct inhibitory subtypes or specific developmental time windows. To devise effective therapeutic strategies, it is crucial to identify the circuit elements relevant to particular symptoms and determine the optimal timing for interventions to achieve meaningful outcomes.

If altered inhibitory tone can selectively affect social and spatial behaviors, it is just as crucial to understand how environmental stress tangles with these microcircuits. At the circuit level, information processing relies on excitatory circuits operating under precise modulation by GABAergic inhibition. In the current Research Topic, Nasretdinov et al. show that CB1 receptor-expressing (CB1R+) INs in layer V of the entorhinal cortex are modulated by stress. These INs regulate excitatory flow within the hippocampal-entorhinal loop while contributing minimally to local feedback inhibition. Consequently, CB1R+ INs in the deep layers of the entorhinal cortex function as a key relay station, translating hippocampal excitation into effective inhibition of cortical pyramidal cells. This provides circuit- and cellular-level mechanisms for linking environmental stress to neuronal activity.

Such sensitivity to both internal and external cues underscores the therapeutic potential of targeting GABAergic elements. In fact, we are beginning to see evidence of how subtle receptor differences can open distinct avenues for pharmacological intervention. Developing effective drugs for brain disorders is one of important missions for neuroscientists. Takasu et al. investigate the effect of allopregnanolone and diazepam, two positive modulators of GABA_A_Rs on abnormal social behaviors and cortical oscillations in social defeat model mice. The authors find that allopregnanolone's selective engagement of extrasynaptic δ-subunit-containing receptors leads to unique changes in circuit oscillations within the basolateral amygdala and medial prefrontal cortex, producing rapid antidepressant-like effects that benzodiazepines cannot replicate. This result echoes a central tenet of our field: subtle differences in receptor composition and localization matter immensely for how interneurons regulate circuit states linked to mood and cognition.

These diverse set of articles coalesce into a powerful message: GABAergic circuits, in all their complexity, underwrite the brain's astonishing adaptability—yet they remain intriguingly susceptible to brain states and stressors. We now see a system in which genetic specification, epigenetic shaping, receptor diversity, and synaptic adhesion cooperate to produce circuits that are robust, plastic, and finely attuned to the ever-shifting computational landscape of the brain. Moving forward, novel approaches combining genetic access, multiomic profiling, and advanced imaging and electrophysiological methods with continuous behavioral monitoring paradigms will provide comprehensive insights linking the molecular logic underlying circuit assembly, cell types, and circuit mechanisms to animal behaviors in health and disease. The NIH SSPSyGene Consortium has taken a bold step toward this ambitious goal by launching a massive initiative to generate and multi-dimensionally profile 100 mouse gene knockouts for neurodevelopmental and psychiatric disorder risk genes—systematically building an accessible catalog of genotypes and phenotypes for open exploration (https://sspsygene.ucsc.edu). The continuous effort on elucidating complexity and specificity inherent in inhibitory neuron biology holds great promise for gaining the power to not only describe cortical circuits in detail, but also to correct their pathological deviations and restore proper function.
